# Quantitative and phylogenetic study of the Deep Sea Archaeal Group in sediments of the Arctic mid-ocean spreading ridge

**DOI:** 10.3389/fmicb.2013.00299

**Published:** 2013-10-04

**Authors:** Steffen L. Jørgensen, Ingunn H. Thorseth, Rolf B. Pedersen, Tamara Baumberger, Christa Schleper

**Affiliations:** ^1^Department of Biology, Centre for Geobiology, University of Bergen, Bergen, Norway; ^2^Department of Earth Science, Centre for Geobiology, University of Bergen, Bergen, Norway; ^3^Department of Earth Sciences, ETH Zurich, Zurich, Switzerland; ^4^Division of Archaea Biology and Ecogenomics, Department of Ecogenomics and Systems Biology, University of Vienna, Vienna, Austria

**Keywords:** Deep Sea Archaeal Group, Marine Benthic Group B, qPCR, deep-sea sediment, geochemistry, iron reduction, manganese reduction, geochemical correlation

## Abstract

In marine sediments archaea often constitute a considerable part of the microbial community, of which the Deep Sea Archaeal Group (DSAG) is one of the most predominant. Despite their high abundance no members from this archaeal group have so far been characterized and thus their metabolism is unknown. Here we show that the relative abundance of DSAG marker genes can be correlated with geochemical parameters, allowing prediction of both the potential electron donors and acceptors of these organisms. We estimated the abundance of 16S rRNA genes from Archaea, Bacteria, and DSAG in 52 sediment horizons from two cores collected at the slow-spreading Arctic Mid-Ocean Ridge, using qPCR. The results indicate that members of the DSAG make up the entire archaeal population in certain horizons and constitute up to ~50% of the total microbial community. The quantitative data were correlated to 30 different geophysical and geochemical parameters obtained from the same sediment horizons. We observed a significant correlation between the relative abundance of DSAG 16S rRNA genes and the content of organic carbon (*p* < 0.0001). Further, significant co-variation with iron oxide, and dissolved iron and manganese (all *p* < 0.0000), indicated a direct or indirect link to iron and manganese cycling. Neither of these parameters correlated with the relative abundance of archaeal or bacterial 16S rRNA genes, nor did any other major electron donor or acceptor measured. Phylogenetic analysis of DSAG 16S rRNA gene sequences reveals three monophyletic lineages with no apparent habitat-specific distribution. In this study we support the hypothesis that members of the DSAG are tightly linked to the content of organic carbon and directly or indirectly involved in the cycling of iron and/or manganese compounds. Further, we provide a molecular tool to assess their abundance in environmental samples and enrichment cultures.

## Introduction

Archaea are widely distributed around the globe and abundant in both the terrestrial and marine realms, where they display a remarkable diversity (Robertson et al., 2005; Schleper et al., 2005; Brochier-Armanet et al., 2011). Although characterized isolates now assigned to this domain have been around for almost 100 years (Klebahn, 1919; Dassarma et al., 2010), the majority remain uncharacterized and are only recognized through their genetic fingerprint (Teske and Sorensen, 2008; Cavicchioli, 2011). These fingerprints, mainly obtained in the form of 16S rRNA genes, contain valuable information about the identity and abundance of organisms, but unfortunately rarely give any clues about their metabolism. This is of particular concern for uncharacterized archaeal (or bacterial) groups with a high abundance and a cosmopolitan distribution, as they might have profound influence on major geochemical cycles. Examples in this respect are anaerobic methanotrophs (ANME) (Boetius et al., 2000; Orphan et al., 2001) and ammonia oxidizing archaea (Konneke et al., 2005; Treusch et al., 2005; Leininger et al., 2006), two abundant groups of archaea whose metabolism only recently have been uncovered.

One of the most prominent archaeal lineages in marine sediments based on 16S rRNA gene surveys is the Deep Sea Archaeal Group (DSAG). No representatives from this group have so far been cultured or otherwise metabolically characterized. The lineage was first described in 1999 by Takai and Horikoshi who obtained 16S rRNA gene signatures from a hydrothermal system, naming the cluster Deep Sea Hydrothermal Vent Crenarchaeotic Group 1 (Takai and Horikoshi, 1999), which was later re-named to DSAG by the same authors (Takai et al., 2001). A few months later Vetriani and colleagues published additional sequence information related to this group from marine sediments obtained in the Atlantic Ocean, assigning the name Marine Benthic Group B (MBG-B) to the cluster (Vetriani et al., 1999). Although several other nomenclatures have been applied to this group over the years (Dong et al., 2006; Robertson et al., 2009) most studies use DSAG, MBG-B or both. Based on 16S rRNA gene information the DSAG form a monophyletic cluster within the Crenarchaeota phylum, and there is currently no evidence supporting an inclusion in the newly defined Thaumarchaeal phylum (Brochier-Armanet et al., 2008; Spang et al., 2010; Pester et al., 2011). In order to resolve a more exact placement of this group in the tree of life, additional genome information is needed.

Signatures of DSAG have been reported from a number of marine habitats, including hydrothermal vents (Takai and Horikoshi, 1999; Reysenbach et al., 2000; Teske et al., 2002; Nakagawa et al., 2005; Hirayama et al., 2007), microbial mats and filaments from different marine settings (Knittel et al., 2005; Omoregie et al., 2008; Robertson et al., 2009; Reigstad et al., 2011), seep systems (Heijs et al., 2007; Dang et al., 2010; Orcutt et al., 2010), deep-sea sediments (Vetriani et al., 1999; Inagaki et al., 2006; Nunoura et al., 2009; Lloyd et al., 2010; Jorgensen et al., 2012), and near-shore and intertidal sediments (Inagaki et al., 2003; Powell et al., 2003; Kim et al., 2005). However, the DSAG is not restricted to the marine environment, as can be concluded from several recent studies reporting their occurrence in inland lakes (Dong et al., 2006; Jiang et al., 2008; Nold et al., 2010; Schubert et al., 2011), a terrestrial cave system (Chen et al., 2009), soil (Kasai et al., 2005), and fresh water iron mats (Kato et al., 2012). Although members of the DSAG apparently have a liberal habitat preference, there is at least one common denominator: they seem to be restricted to anaerobic or micro-aerophilic environments. Furthermore, it has been suggested that they prefer moderately saline and alkaline conditions (Jiang et al., 2008). DSAG signatures are often part of the dominating archaeal 16S rRNA gene pool in marine sediments (Orcutt et al., 2011). In an attempt to quantify their absolute abundance in environmental samples, Knittel and co-workers successfully designed and applied specific FISH probes, and were able to visualize small coccoid-shaped cells, but due to their small size (0.2–0.4 μm) they evaded enumeration (Knittel et al., 2005).

Despite the lack of cultured representatives and metagenomic information related to the DSAG, several indirect measures gave rise to hypotheses about their metabolism. DSAG are very often found in methane-producing environments and thus have been speculated to be involved in methane cycling (Knittel et al., 2005; Biddle et al., 2006; Inagaki et al., 2006; Sorensen and Teske, 2006), and perhaps also linked to the sulfur cycle (Inagaki et al., 2006). However, stable isotope and subsequent lipid analysis have shown that organic carbon rather than methane-derived carbon is being assimilated in the cells (Biddle et al., 2006). Furthermore, DSAG are not restricted to the sulfate/methane transition zone and are found in environments that are sulfate limited, e.g., fresh water systems and sulfate depleted marine sediments (Teske and Sorensen, 2008). These observations make it unlikely that DSAG performs the type of sulfate-dependent anaerobic methane oxidation known from the archaeal ANME lineages (Boetius et al., 2000), but rather point to a heterotrophic or mixotrophic life style. Such a life style was recently supported by our own studies, in which we proposed that the DSAG could be involved in the iron cycle, using iron oxide as a terminal electron acceptor while oxidizing organic carbon (Jorgensen et al., 2012). This proposal was based on a tight correlation between the relative abundance of DSAG 16S rRNA genes in deep-sea sediment and concentrations of organic carbon and iron oxide. In addition a clear covariance with dissolved iron was observed. However, this covariance was not significant and it was speculated that it could be due to limited data points.

In order to investigate the occurrence and possible metabolism of the DSAG in higher resolution we study here two sediment cores retrieved from the Norwegian-Greenland Sea. The cores were taken within a radius of 1 km from the area in the Arctic Mid-Ocean rift valley where our previous study was conducted. In total we investigate and analyze 52 deep-sea sediment horizons with respect to the occurrence and abundance of DSAG 16S rRNA genes by applying qPCR with newly designed primers. These quantitative results are evaluated in the context of 30 different geochemical parameters from both the solid and interstitial phase in order to examine potential covariance patterns.

We also revisit the phylogeny of this group in order to uncover potential biogeographic distribution patterns.

## Materials and methods

### Sampling sites and shipboard sampling

A Calypso Corer System (piston coring) was employed approximately 15 km north of the Loki's Castle vent field in the rift valley of the Arctic Mid-Ocean Ridge and successfully recovered ~11.2 m of sediment (core PC15, 73°45.38'N - 8°27.31'E, 3236 mbsl). In addition, a 2-m long gravity core (core GC14, 73°45.79'N - 8°27.84'E, 3283 mbsl) was retrieved less than 1 km from core PC15 and approximately 100 m from a previously described gravity core (GC12) (Jorgensen et al., 2012). The sample area has been described earlier (Pedersen et al., 2010; Jorgensen et al., 2012). The retrieved cores were immediately split in halves and sampling of porewater and sediment for geochemical and microbial analyses were immediately conducted onboard the ship. The samples for microbial studies were collected at depth intervals of 30 cm throughout the core length using sterile 10 ml syringes. The sediment was either processed immediately or snap-frozen in liquid nitrogen before storage at −80°C. Porewater was extracted with Rhizon samplers from nearly identical depths as the microbial samples and split into subsamples for immediate shipboard and later on-shore analysis.

### DNA extraction

DNA was extracted from approximately 0.5 g of sediment using FastDNA® spin kit for soil in conjunction with the FastPrep®-24 instrument (MP Biomedicals) following manufacturer's protocol applying the poly-A modification described by Hugenholtz and colleagues (Hugenholtz et al., 1998).

### Design of DSAG specific primers, PCR and cloning

Two DSAG 16S rRNA gene specific forward primers DSAG 384f (5'TGTGACGGGGTTACCCAA3') and DSAG 535f (5'ACCAGCTCTTCAAGTGG3') were designed using the ARB work package in conjunction with the Silva database release 104 (Ludwig et al., 2004; Pruesse et al., 2007) and tested both *in silico* and *in situ*. The primers have full match to 76 and 94% of all DSAG sequences in the Silva database Release 111, respectively (931 sequences) and no match outside the target group. Allowing one mismatch increased the percentages to 84 and 99%, respectively, however the DSAG 535f then matched 119 sequences outside the DSAG target group, mainly within the Miscellaneous Crenarchaeal Group (79 sequences) and the Marine Hydrothermal Vent Group (36 sequences). Both primers were used in conjunction with the archaeal specific Arc908r primer (5'CCCGCCAATTCCTTTAAGTT3') (Jorgensen et al., 2012). To verify primer specificity and optimize PCR conditions a thermal gradient PCR was executed, each reaction (25 μl) containing 1× Qiagen HotStarTaq master mix (Qiagen), 0.8 μM of each primer and 1 μl template. As template, DNA extracted from various environmental sources was used: DSAG-positive sediment samples (positive control), seawater, terrestrial hot springs, Arctic soil as well as genomic DNA from several bacterial and archaeal pure cultures (negative controls). Optimal thermal conditions were found to be 15 min at 95°C (polymerase activation), then 35 cycles of 95°C/30 s, 59°C/30 s, 72°C/45 s, final extension at 72°C for 10 min before cooling to 4°C. Gel electrophoresis was performed to visualize correct amplicon size.

In order to verify primer specificity, amplicons obtained with the DSAG 384f and the Ar908r primers were cloned (TOPO TA system, Invitrogen), and inserts from 60 randomly picked clones with correct insert length sequenced (ABI 3730xl sequencer, Applied Biosystems). All of the sequenced clones were checked for chimeras with Chimera Check (Cole et al., 2009) and their taxonomic affiliation to DSAG verified using the web-based Classification Resources for Environmental Sequence Tags (CREST) (Lanzen et al., 2012). No chimeric sequences were observed and all sequences affiliated with DSAG. The 60 clones represent five different OTUs (97% cut-off) and all cluster within one group in the Gamma lineage (marked with asterisk in Figure 3). Sequences are deposited at the NCBI under accession numbers KF578027–KF578075.

### Real-time quantitative PCR (qPCR)

**Figure 1:**
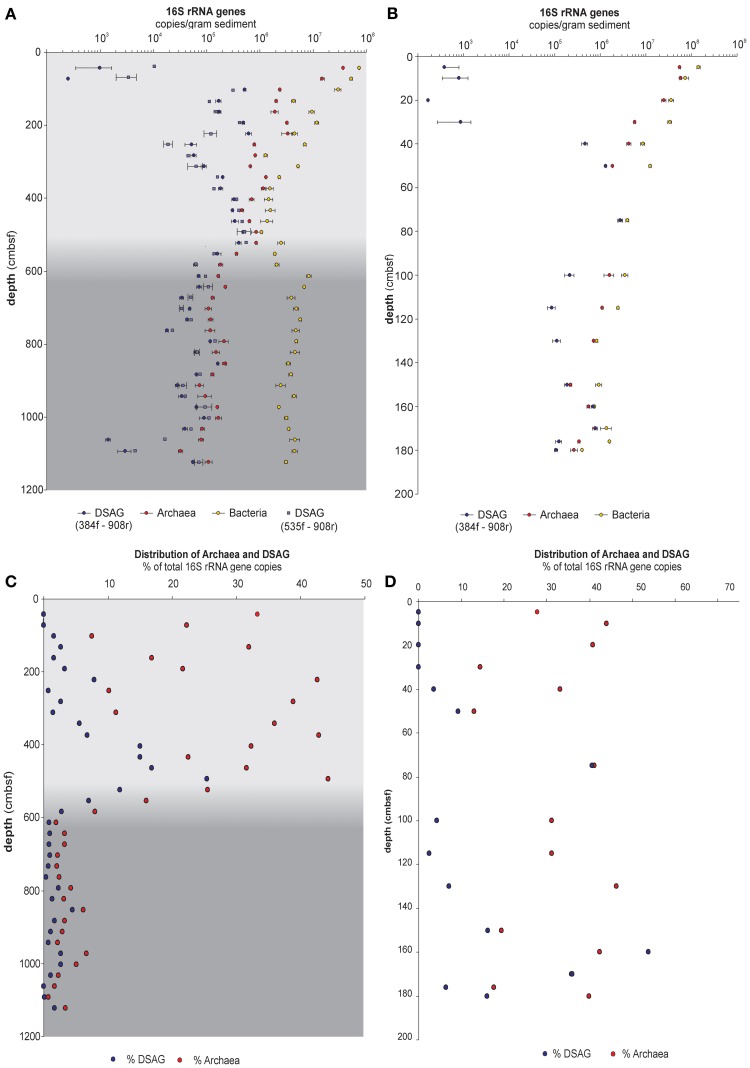
**QPCR data from sediment core PC15 and GC14. (A)** Absolute 16S rRNA gene copies per gram sediment of bacteria, archaea, and DSAG in core PC15. The latter estimated with two different forward primers, 384f and 535f. Error bar indicates standard deviation of triplicate samples. **(B)** Data from core GC14 but otherwise identical to panel **(A)**. **(C)** Proportion of Archaea and DSAG 16S rRNA gene copies out of the total abundance (Archaea + Bacteria) in PC15. Values are given in percent of total. **(D)** Data from GC14 but otherwise identical to panel **(C)**. Gray color shading indicates a lithological transition in the sediment at approximately 5.5 mbsf, which marks the onset of a debris flow.

DSAG 16S rRNA genes were quantified using DSAG 384f and the archaeal-specific Arc908r. Each reaction (20 μl) contained 1× QuantiTech SybrGreen PCR master mixture (Qiagen), 0.8 μM of each primer and 1 μl template DNA. Thermal conditions were 15 min at 95°C (polymerase activation), then 35 cycles of 95°C/30 s, 59°C/30 s, 72°C/45 s, with plate read after each cycle. A purified PCR amplicon obtained with M13 primers from a DSAG positive clone was used as standard. The R^2^ values for the standard curves were >0.99 and the estimated amplification efficiencies were between 93 and 99%. As the DSAG 384f primer has a mismatch to sequences in the Beta cluster (Figure 3) the qPCR results from PC15 were verified applying the broader targeting DSAG 535f primer on all samples under the same conditions as applied with the DSAG 384f; both primers gave similar results (Figure 1A).

Archaeal 16S rRNA genes were quantified using the prokaryotic primer Uni519F (5'CAGCMGCCGCGGTAA3') (Ovreas et al., 1997) and the archaeal-specific primer Arc908r (5'CCCGCCAATTCCTTTAAGTT3'), as described elsewhere (Jorgensen et al., 2012). The R^2^ value for all standard curves was >0.99 and the estimated amplification efficiency between 92 and 98%.

Bacterial 16S rRNA genes were quantified using the bacterial-specific primer bac341f (5'CCTACGGGWGGCWGCA3') (Jorgensen et al., 2012) and the prokaryotic 519r (5'TTACCGCGGCKGCTG3') (Ovreas et al., 1997), as described elsewhere (Jorgensen et al., 2012). The R^2^ values for the standard curves was all >0.99 and the estimated amplification efficiency ranged between 100 and 105%.

All qPCR experiments were performed with the Step-OnePlus real-time PCR system (Applied Biosystems) using SYBRGreenI as fluorescent dye. To confirm product specificity, melting curve analyses were performed after each run for all experiments and each qPCR setup contained samples, standard series, negative controls and blanks in triplicates.

Primer specificity is always of high concern in this type of analysis and although no guarantee of the exact performance of a primer pair and their coverage in any given environmental sample can be given, it is worth noting the *in silico* performance. Both the archaeal and bacterial primer pair matches 89% in the RDP database (release 10, update 32) without mismatch and without match outside the target group. The numbers are 97% and 98%, respectively, allowing one mismatch. However, this also allows match outside the target group, although limited (below 1%).

### Phylogenetic analysis

The phylogeny of the DSAG was evaluated based on published 16S rRNA gene sequences (>900 bp) available in the Silva database release 111 (Pruesse et al., 2007), if they had sequence information between position 274 and 957 (*E. coli* numbering). This left 497 sequences and 647 valid columns for phylogenetic calculations. *Archaeoglobus* was used as outgroup (13 sequences). The displayed consensus tree is based on Neighbor Joining (NJ) with Felsenstein correction (Felsenstein, 1985) and Maximum Likelihood (RaxML and PhyML) algorithms (Guindon and Gascuel, 2003; Stamatakis et al., 2005). All clusters were moved back to a branching point supported by all three algorithms (Figure 3). The topology of the tree was verified by all three algorithms and shows three major monophyletic DSAG lineages, termed Alpha, Beta, and Gamma. DSAG, high throughput 454 amplicon sequences from our previous study (Jorgensen et al., 2012) were clustered into 18 OTUs (97% cut-off) and added to the tree using the parsimony tool in the ARB work package and their positions are marked in Figure 3. We note that more than 60% of all published sequences in the Silva database (SSUref 111) originated from a single habitat, the hypersaline ponds, Guerrero Negro in Baja California, Mexico (Ley et al., 2006; Jahnke et al., 2008; Robertson et al., 2009). Further, we identified a fourth cluster of sequences designated DSAG in the Silva database. However, this cluster was only distantly related to any of the other three clusters (<82.5% identical) and is not monophyletic with the DSAG, in our analysis. In addition they all had unsettling low pintail values. Thus, the following 11 sequences were excluded from the final analysis; EU731634, EU731636, EU731638–39, EU731641, EU731643–45, EU731649, AY861998, AY861996.

### Geochemical measurements

Part of the sediment samples used in the microbial analysis was also analyzed for the content of total organic carbon (TOC) by coulometric titration (CM5012 CO_2_ Coulometer). Samples were dried overnight and total inorganic carbon (TIC) and total carbon (TC) were analyzed. The TOC content was estimated by subtracting the two parameters, leading to an error of ±0.05%C for TOC content. Major oxides (Na_2_O, MgO, Al_2_O_3_, SiO_2_, P_2_O_5_, K_2_O, CaO, TiO_2_, MnO_2_ and Fe_2_O_3_), were analyzed in 47 sediment samples (11 from GC14 and 36 from PC15) by x-ray fluorescent (XRF) spectroscopy (Philips PW1404). Further, porewater chemistry of 50 sediment horizons (13 from GC14 and 37 from PC15) was analyzed for major anions (Cl, SO_4_^2−^) by ion chromatography (Metrohm) and for major and trace elements (Na, Mg, K, Ca, Si, Sr, Ba, Fe^2+^, Mn^2+^) by Inductively Coupled Plasma Optical Emission Spectrometry (Elemental). In addition, aliquots of the same porewater samples were analyzed shipboard for H_2_S, NH_4_^+^, NO_3_^−^, NO_2_^−^, and PO_4_ by colorimetric methods using a continuous flow analyzer (Seal), for alkalinity by an autotitrator (Metrohm), and for pH using a portable meter (Metrohm). Methane measurements were carried out for PC15 at 2 m intervals by adding 5 ml of sediment to a 140 ml syringe immediately after the cores were split. 1.2 M NaCl solution with sodium azide (0.1%) was added up to 100 ml and additional 40 ml helium gas was added before shaking. The syringe was left for at least half an hour at room temperature to reach equilibration between the gas and water phase before the headspace was analyzed by a gas chromatograph (SRI 8610C) equipped with a flame ionization detector (FID).

## Results

### Quantitative PCR

**Figure 2:**
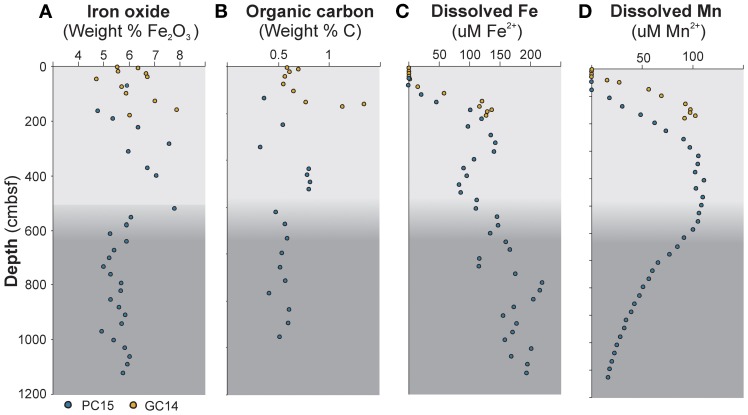
**Geochemical depth profiles of sediment cores PC15 (blue circles) and GC14 (yellow circles). (A)** Iron oxide content in the solid phase. **(B)** Total organic carbon content (TOC). **(C)** Dissolved iron in the porewater and **(D)** dissolved manganese in the porewater. Gray color shading indicates a lithological transition in the sediment at approximately 5.5 mbsf, which marks the onset of a debris flow.

We quantified the 16S rRNA genes of Bacteria, Archaea, and DSAG in a total of 52 sediment horizons from two cores (37 from PC15 and 15 from GC14). Both investigated cores had been retrieved in an area where a previous study has suggested a high abundance of DSAG and an interesting compressed porewater profile with elevated iron concentrations (Jorgensen et al., 2012). The results from the qPCR measurements show a depth profile with archaeal 16S rRNA gene copy numbers/gram of sediment estimated to be on the order of 10^8^ in the upper layers and declining by approximately two orders of magnitude within the first 2 m. This trend was observed in both the piston core (Figure 1A) and the shorter gravity core (Figure 1B). Both primers specifically designed for DSAG (384F and 535F) gave similar results (Figure 1A). The ratio between archaeal and bacterial 16S rRNA gene copies fluctuated considerably between adjacent layers and was between 0.01–0.44 in PC15 and 0.18–0.46 in GC14, (Figures 1C,D, respectively). It is interesting to note that the average proportion of Archaea in the microbial population was 0.27 in the upper half of the piston core (~5.5 mbsf) whereas in the lower part it decreased to 0.03 (Figure 1C). This change in ratio coincided with profound changes in the geochemical composition of the sediment, as indicated by a transition in the concentration of several of the major oxides. This is also reflected in the many tight correlations observed between the relative abundance of archaeal and bacterial 16S rRNA gene copies and different oxides, e.g., SiO_2_, MgO, and Na_2_O (supplementary table 1). DSAG was below or just above detection limit in the presumed oxic top layers (0–100 cmbsf and 0–40 cmbsf in PC15 and GC14, respectively), whereas they were well above detection limit in all anoxic horizons (Figures 1A,B). Both their absolute and relative abundances of 16S rRNA genes fluctuated substantially between adjacent horizons, as were also observed for Bacteria and Archaea and for the geochemical parameters (Figures 1, 2). The relative abundance, measured as the percent of DSAG 16S rRNA gene copies of the total number (Archaea plus Bacteria), varied between 0–25% in PC15 and 0–54% in GC14 (Figures 1C,D, respectively).

### Geochemical properties

A range of geochemical components was measured in order to investigate potential correlations with the microbial data in general and with the DSAG in particular. From the interstitial phase, the following major microbial electron donors and acceptors were analyzed: Fe^2+^, Mn^2+^, NH_4_^+^, NO_3_^−^, H_2_S, SO_4_^+^, and CH_4_. In addition, 10 other porewater constituents were measured, none of them known to be of direct relevance to microbial metabolism (supplementary table 1). Nitrate, manganese (Figure 2D) and ammonium displayed typical diagenetic depth profiles in both cores. Dissolved iron was detected already in the manganous zone and the concentrations (21–220 μM in PC15 and 2–136 μM in GC14) increased with depth in a fluctuating manner (Figure 2C). Methane and sulfide were below detection, the latter despite a moderate rate of sulfate reduction as indicated by decreasing sulfate concentrations with depth, likely as a result of the chemical reaction with dissolved iron and subsequent precipitation. Further, the concentrations of the two potential electron acceptors manganese and iron oxide were measured, along with eight other major oxides, in the solid phase. The content of iron oxide (Fe_2_O_3_) in the solid sediment was in the range of 4.72–7.88 weight % in GC14 and 4.77–7.8 weight % in PC15 (Figure 2A). Manganese oxide (MnO_2_) was 0.07–0.3 weight % in PC15 and the values ranged between 0.05 and 1.3 weight % in GC14. In addition, TOC content was measured and varied between 0.3 and 0.8 weight % in the piston core (PC15) and between 0.6 and 1.3 in the shorter gravity core (GC14) (Figure 2B). All values were comparable with what has previously been reported from this area (Jorgensen et al., 2012).

### Phylogenetic analysis

**Figure 3:**
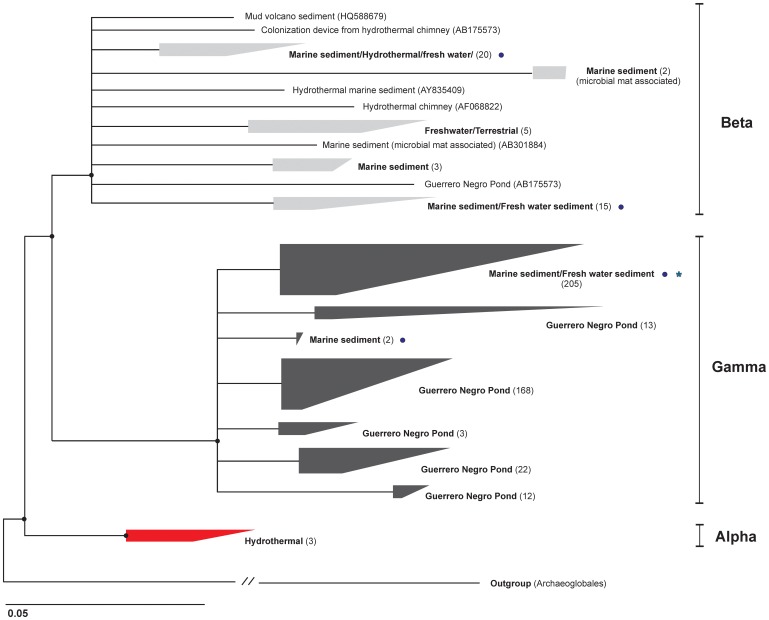
**Phylogenetic tree of the Deep Sea Archaeal Group.** The tree displays a consensus of topologies from neighbor joining and maximum likelihood algorithms. The number in parentheses is the number of sequences used to build the tree within that cluster. The asterisk indicates the group where all the sequences from the 60 clones obtained in this study affiliate. Circles mark where the 18 OTUs (7385 sequences) obtained in our previous study (Jorgensen et al., 2012) cluster. Of these, 99% fall within the Gamma lineage.

We used a total of 497 published DSAG sequences (≥900 bp) and 647 valid columns to calculate phylogenetic trees by different algorithms. The tree presented in Figure 3 is a consensus supported by neighbor joining and maximum likelihood (RaxML and PhyML). The analysis revealed three distinct monophyletic lineages within the DSAG; Alpha, Beta and Gamma. The Alpha cluster was the deepest branching within the group and consisted exclusively of sequences retrieved from hydrothermal environments. The Beta cluster contained sequences from a diverse range of habitats, including lake and terrestrial cave sediments and water, sediments from marine hydrothermal areas, mud volcanoes, and salt marsh environments as well as from the Guerrero Negro Pond system. More than half (57%) of the sequences within the Gamma cluster originated from the Guerrero Negro Pond 4 and the remaining sequences have been recovered from a diverse range of marine settings. All the sequences in the Gamma cluster contained a 6 bp deletion around *E. coli* position 415 and 427, that none of the alpha and only few beta-affiliated sequences had.

The entire group of DSAG was represented by 185 OTUs when using all available sequences in the Silva database (SSUref 111) (97% cut-off and 665 bp minimal length), whereof 86 OTUs are represented in the phylogenetic tree showing all published sequences from the same database. The minimum sequence similarity within the Alpha, Beta and Gamma clusters, over the 665 bp used, was 95%, 83% (two sequences, otherwise 88%) and 90%, respectively. The 454 pyrosequenced 16S rRNA gene amplicons obtained with general prokaryotic primers in our previous study showed that 82% of all our DSAG sequences (7385 in total) fall within one group in the gamma lineage and only 1% cluster within the Beta lineage (Jorgensen et al., 2012) (marked with circles in Figure 3). Based on these results we conclude that the Beta lineage likely constitutes a minor part of the DSAG community in these cores.

### Comparison between relative abundance data and geochemistry

In order to test for any significant covariance between the relative abundance of DSAG marker genes and geochemical data we log-transformed the data and performed a simple Pearson correlation coefficient analysis (supplementary table 1). In the analysis we included data from the two cores described here and an additional gravity core (GC12) obtained in 2008, where the geochemistry and DSAG abundances (based on 454 pyrosequencing of 16S rRNA gene amplicons) have been described previously (Jorgensen et al., 2012).

**Figure 4:**
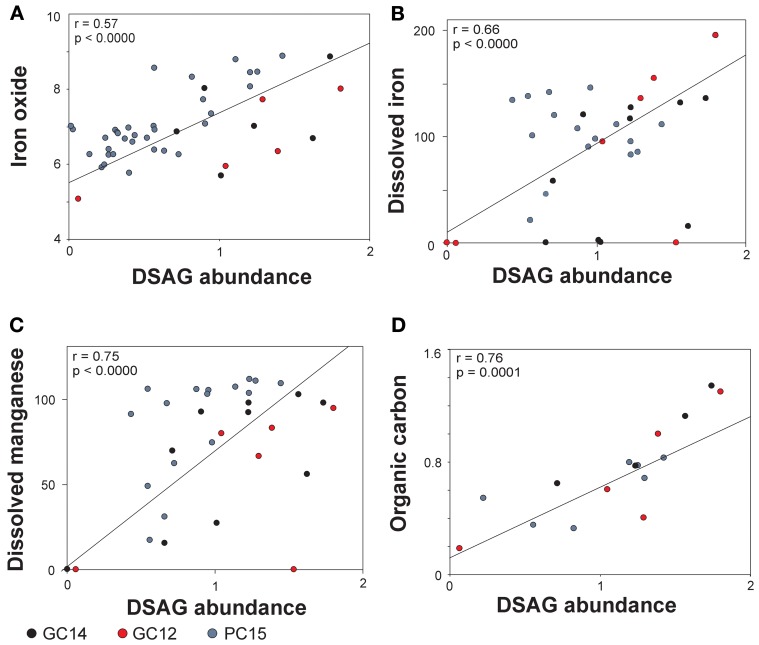
**Pearson correlations between abundance of DSAG in core GC14, PC15 and GC12 and the geochemical parameters. (A)** Iron oxide, **(B)** dissolved iron, **(C)** dissolved manganese and **(D)** total organic carbon. The correlations with dissolved iron and manganese are based on a subset of data, where the lower part of PC15 is excluded (see supplementary table 1). DSAG abundance is given as % DSAG 16S rRNA gene copies of total 16S rRNA gene copies (log).

The results show a significant correlation (*p* ≤ 0.0001) between the relative abundance of DSAG 16S rRNA genes and the concentration of solid iron oxide (*r* = 0.57 and *p* < 0.0000, Figure 4A). None of the other major oxides in the solid phase revealed any significant correlation (supplementary table 1). The concentration of dissolved iron (Fe^2+^) in the porewater covaried with the abundance of DSAG 16S rRNA genes, although not significantly (*r* = 0.34 and *p* = 0.01, supplementary table 1A). However, due to the sudden change in the ratio between Archaea and Bacteria and the concurrent change in geochemistry at approximately 5.5 mbsf in PC15 it seems clear that the upper and lower core segments have very different geochemical and/or geophysical properties. We therefore executed the analysis again, excluding all data points below this depth. This resulted in a strong increase in the correlation (*r* = 0.66 and *p* < 0.0000, Figure 4B, supplementary table 1B). Further, a significant correlation was also observed with dissolved manganese (Mn_2_^+^) in the porewater, again only when the lower part of PC15 was excluded from the analysis (*r* = 0.75 and *p* < 0.0000, Figure 4C, supplementary table 1B). Besides the three geochemical variables mentioned above, the DSAG 16S rRNA gene copy abundance co-varied with the content of organic carbon (*r* = 0.76 and *p* < 0.0001, Figure 4D). Notably, no significant correlation with depth, pH, nitrate or sulfate was observed. Further, none of the additional measured porewater constituents co-varied significantly with DSAG 16S rRNA gene abundance, although ammonium showed a strong correlation (*r* = 0.59, *p* = 0.0002, supplementary table 1). With respect to the relative abundance of archaeal and bacterial 16S rRNA copies they both correlated significantly with a number of the measured parameters, although none known to be potential electron acceptors or donors (supplementary table 1).

## Discussion

Previous findings had suggested that sediments in the study area have a compact geochemical depth profile with relatively shallow sulfate reduction and elevated concentrations of dissolved iron in combination with a high abundance of DSAG marker genes (Jorgensen et al., 2012). This abundance was linked to the content of both organic carbon and iron oxide and additionally showed a positive trend with dissolved iron, although not significant. Along with the special geochemical nature of the sediments, this motivated us to collect a new replicate gravity core (GC14) and a longer piston core from the same area (PC15), in order to validate some of the previous observations by specific assays and with a higher spatial resolution.

Our qPCR results from these cores support a high abundance of DSAG 16S rRNA genes, constituting up to 100% of all archaeal an estimated 50% of the total microbial community in specific horizons (Figures 1C,D). The geochemical results suggest that we recovered five geochemical redox zones; oxic, nitrogenous, manganous, ferruginous, and sulfidic (based on sulfate depletion), but the zonation appeared to be less compressed than in the cores previously studied (Jorgensen et al., 2012). Further, the high level of dissolved iron in the porewater was confirmed (Figure 2C) and suggests active microbial iron reduction from the upper anoxic horizons and throughout the length of both cores. We show here that the content of iron oxide in the measured sediment horizons, as the only one of the major oxides, is significantly correlated to the relative abundance of DSAG 16S rRNA genes (Figure 4A, supplementary table 1).

The ability to utilize various forms of iron oxide, such as poorly crystalline iron oxides, as terminal electron acceptor, is a widespread trait in Bacteria and Archaea (Lovley and Phillips, 1988; Lovley et al., 2004; Weber et al., 2006). Iron is released into the porewater upon microbial reduction of iron oxide and in our study the concentration of dissolved iron increased with depth displaying a fluctuating pattern, presumably due to differences in reduction and/or oxidation rate in specific sediment horizons. We were able to correlate this pattern with the relative abundance of DSAG (Figure 4B, supplementary table 1B) but only when we excluded data from the lower half of core PC15, in which the relative proportions of Archaea and Bacteria changed dramatically (Figure 1B). This change in ratio coincides with the transition from a fine silty material to a coarser grained homogenous sediment, deposited by a extensive glaciogenic debris flow that prevail throughout the length of the core. This is likely to cause drastic changes in the geophysical and geochemical properties of the sediment, as can also be seen from the geochemical data. When excluding the lower half we also notice a significant covariance with dissolved manganese, which might suggest that manganese oxide, likewise is a potential electron acceptor for this group, or specific members of it (supplementary table 1B). Such a scenario would not be surprising as most of the characterized iron reducing strains also have the capability to reduce manganese (Nealson and Saffarini, 1994; Lovley et al., 2004).

Notably, none of the other 30 geochemical and geophysical parameters measured showed any significant correlation with the DSAG 16S rRNA gene abundance (supplementary table 1). However, relative abundance of ribosomal DNA is not necessarily a good measure for activity and this has to be kept in mind when evaluating these data.

Various forms of organic carbon can be used as electron donors by iron reducing microorganisms, and indirect evidence for an organotrophic lifestyle among the DSAG was provided by stable isotope experiments and subsequent lipid analysis by Biddle and co-workers (Biddle et al., 2006). This suggestion was supported by our previous observed correlation between the abundance of DSAG and organic carbon (Jorgensen et al., 2012). Here we verify this correlation for both cores (Figure 4D, supplementary table 1). However, beside organic carbon, both H_2_ (Lovley et al., 1989) and ammonia (Clement et al., 2005) have been discussed as possible electron donors for iron reducers, although the latter still lacks final evidence. In addition, certain sulfate reducing and methanogenic microorganisms have also been reported to have the ability to reduce iron oxide and although this reduction is without the benefit of growth, this could be another explanation for the observed correlations (Roberts, 1947; Lovley and Phillips, 1986; Coleman et al., 1993; Lovley et al., 1993; Bond and Lovley, 2002).

Beside the obvious possibility that members of the DSAG use organic carbon as electron donor, fermentation where iron oxide is used as an electron sink could also explain the observed correlations in our study. However, it has been demonstrated that only a very small part of the reducing equivalents are shuttled through to iron oxide (Lovley and Phillips, 1986). Further, it has been speculated that microbially mediated reduction of iron could be coupled to the anaerobic oxidation of methane, a process that theoretically yields more energy than the coupling between methane and sulfate (Caldwell et al., 2008; Beal et al., 2009). Methane measurements were conducted throughout the length of PC15. However, all measurements were below detection and thus no link between DSAG and methane could be inferred. Further, although unlikely, we cannot rule out that the observed covariance with organic carbon is due to underlying correlations, such as between hydrogen production and organic carbon concentration, implying that the DSAG perform iron and/or manganese reduction coupled to hydrogen oxidation. Considering the above-mentioned hypotheses in the light of our data, we think the most plausible metabolism for DSAG seems to be oxidation of organic carbon coupled to the reduction of iron oxide and/or manganese oxide. However, these correlations, as all correlation analysis should be evaluated with caution, as causality cannot be inferred based on this type of data alone. Thus, one or several underlying parameters might be the true reason why significant correlations are observed.

The phylogenetic analysis of the DSAG indicates no apparent habitat-specific clustering of sequences as opposed to findings for other archaeal groups such as the Korarchaeota and the Marine Group I (Auchtung et al., 2006; Durbin and Teske, 2010; Reigstad et al., 2010; Jorgensen et al., 2012). However, sequences in the deepest branching DSAG group (Alpha) are exclusively of hydrothermal origin, suggesting a hydrothermal origin of the entire group, as also previously argued (Reysenbach et al., 2000; Teske et al., 2002). In this context, it is noteworthy to mention that all known archaeal groups able to perform dissimilatory iron reduction are thermophilic (Weber et al., 2006). It is intriguing to speculate that this trait might have been passed on from thermophilic DSAG lineages to other members of this group that evolved and adapted to a mesophilic lifestyle.

An interesting finding in our qPCR study is the sudden ten-fold decrease in the ratio between archaeal and bacterial 16S rRNA gene copies (from average 0.27 to less than 0.03) in core PC15. Overall the total copy numbers stayed relatively stable from approximately 2 m below surface and throughout the core (10^7^ copies/gram sediment), which suggest that the available energy is unchanged and sustains the same number of organisms. However, caution has to be taken in the interpretation as differences between copy numbers in microbial groups, the presence of extracellular DNA, along with the biases that PCR amplification and quantification can inflict means that copy numbers not necessarily reflect neither cell abundance nor activity. The underlying reason for the observed decrease in the proportion of Archaea is not known, but we note the following: The change is gradual over approximately 1 m. It is tightly coupled to changes in the geochemical composition of the sediment, reflecting a shift in the lithology from fine silty sediment to a coarser grained homogenous glaciogenic debris flow deposit. Probably, one or more bacterial taxa have an advantage under the geophysical and geochemical conditions prevailing below 6 m sediment depth, which enables them to out-compete others. We also note that the relative 16S rRNA gene abundance of both bacteria and archaea co-varies with several of the measured geochemical parameters, although none of them known to be of importance in microbial redox reactions. This could suggest that the abundances of the two domains are tightly coupled to the geochemical nature of the sediment.

In sum we find that the DSAG is a dominating microbial group in these sediments and that their relative abundance is tightly linked to both the concentration of solid iron oxide and dissolved iron in the interstitial water, a strong indication of iron reducing capabilities. Further, the data indicate that the group also could be involved in the manganese cycle. We also verify that DSAG 16S rRNA gene abundance is correlated to organic carbon, an observation that falls in line with the earlier hypothesis that DSAG are organotrophic organisms. Despite these indications it is clear that further studies are needed to clarify if the correlations are of a direct or indirect nature. For future efforts to obtain enrichments and ultimately pure cultures of this group we provide a qPCR protocol to monitor the abundance of DSAG.

## Conflict of interest statement

The authors declare that the research was conducted in the absence of any commercial or financial relationships that could be construed as a potential conflict of interest.
