# Supplementary material for: Quantitative and phylogenetic study of the Deep Sea Archaeal Group in sediments of the Arctic mid-ocean spreading ridge
Source: Front Microbiol. 2013 Oct 4;4:299. doi: 10.3389/fmicb.2013.00299 (PMC3790079; doi:10.3389/fmicb.2013.00299)
Supplement: Supplementary file 1 [file DataSheet1.PDF]

Supplementary table 1: Pearson correlations between relative 16S rRNA gene abundance (qPCR) and geochemical and geophysical parameters.Significant correlations ($p < 0.0000$) are marked in red. **A**) Shows data when all data-points are used and **B**) when the lower half of core PC15 is excluded. N is number of samples used in the calculation.

A

		DSAG		Archaea		Bacteria		
		P values	R values	P values	R values	P values	R values	n
Solid phase	Na_2O	0.7835	0.04	0.0000	0.57	0.0015	-0.45	46
	MgO	0.0033	0.42	0.0000	0.72	0.0000	-0.65	46
	Al_2O3	0.0004	0.50	0.0003	0.51	0.0003	-0.51	46
	SiO_2	0.1477	-0.22	0.0000	-0.57	0.0011	0.47	46
	P_2O_5	0.0439	0.30	0.0072	0.39	0.0028	-0.43	46
	K_2O	0.0003	0.51	0.0001	0.53	0.0001	-0.53	46
	CaO	0.0957	-0.25	0.9855	0.00	0.5466	0.09	46
	TiO_2	0.0005	0.49	0.0000	0.60	0.0000	-0.57	46
	MnO	0.0372	-0.29	1.0000	0.00	0.9520	0.01	52
	Fe_2O_3	0.0000	0.57	0.0025	0.41	0.0944	-0.23	52
	Depth	0.0432	-0.30	0.0000	-0.58	0.0072	0.39	55
	TOC	0.0000	0.77	0.0801	0.32	0.0651	-0.33	31
Interstitial phase	pH	0.0011	-0.43	0.0001	-0.50	0.0000	0.66	55
	Alkal	0.8187	-0.03	0.0000	-0.69	0.0003	0.47	55
	H_2S	-	-	-	-	-	-	55
	NH_4^+	0.2103	-0.17	0.0010	-0.50	0.0004	0.46	55
	NO_3^-	0.0353	-0.28	0.0241	0.30	0.1107	-0.22	55
	SO_4	0.2429	0.16	0.0009	0.51	0.0018	-0.41	55
	Mn	0.0002	0.51	0.7105	-0.05	0.7826	-0.04	55
	Fe	0.0101	0.34	0.0001	-0.50	0.2369	0.16	55
	Si	0.6453	-0.06	0.0000	-0.60	0.0048	0.37	55
	B	0.0280	-0.30	0.0052	-0.37	0.0000	0.59	55
	PO_4	0.0119	-0.34	0.0002	-0.48	0.0005	0.45	55
	Cl	0.1448	-0.20	0.6342	-0.07	0.9442	0.01	55
	Na	0.0376	0.28	0.0000	0.57	0.0000	-0.57	55
	Mg	0.0575	0.26	0.0000	0.63	0.0000	-0.59	55
	K	0.0084	0.35	0.0000	0.61	0.0000	-0.71	55
	Ca	0.0509	0.26	0.2440	0.16	0.0005	-0.45	55
	Sr	0.2049	0.17	0.4114	-0.11	0.0666	-0.25	55
	Ba	0.5742	-0.08	0.0617	-0.25	0.0002	0.47	55

B

		DSAG		Archaea		Bacteria		
		P values	R values	P values	R values	P values	R values	n
Solid phase	Na_2O	0.0015	-0.57	0.9384	0.02	0.9215	-0.02	28
	MgO	0.2743	0.21	0.0064	0.50	0.0170	-0.45	28
	Al_2O3	0.0010	0.59	0.0034	0.53	0.0143	-0.46	28
	SiO_2	0.5329	0.12	0.3808	-0.17	0.5090	0.13	28
	P_2O_5	0.3894	0.17	0.4054	0.16	0.1337	-0.29	28
	K_2O	0.0111	0.47	0.0034	0.53	0.0117	-0.47	28
	CaO	0.0218	-0.43	0.1346	-0.29	0.1327	0.29	28
	TiO_2	0.0035	0.53	0.0020	0.56	0.0139	-0.46	28
	MnO	0.0064	-0.46	0.1758	-0.24	0.5148	0.12	34
	Fe_2O_3	0.0069	0.45	0.1784	0.24	0.6859	-0.07	34
	Depth	0.0895	0.33	0.1469	0.28	0.8674	-0.03	37
	TOC	0.0006	0.66	0.3032	0.23	0.2979	-0.23	22
Interstitial phase	pH	0.0401	-0.34	0.0023	-0.48	0.0000	0.61	37
	Alkal	0.0000	0.63	0.9933	0.00	0.9706	0.01	37
	H_2S	-	-	-	-	-	-	37
	NH_4^+	0.0002	0.59	0.8551	0.03	0.3720	-0.15	37
	NO_3^-	0.0030	-0.47	0.3012	0.17	0.6466	-0.08	37
	SO_4	0.0104	-0.41	0.8858	-0.02	0.4722	0.12	37
	Mn	0.0000	0.75	0.9221	0.02	0.5538	-0.10	37
	Fe	0.0000	0.66	0.8807	0.03	0.4639	-0.12	37
	Si	0.0282	0.36	0.9097	0.02	0.7669	-0.05	37
	B	0.1815	-0.22	0.0160	-0.39	0.0002	0.57	37
	PO_4	0.1068	-0.27	0.0066	-0.44	0.0248	0.37	37
	Cl	0.2563	-0.19	0.9980	0.00	0.8018	-0.04	37
	Na	0.7775	0.05	0.1397	0.25	0.0078	-0.43	37
	Mg	0.7753	-0.05	0.1955	0.22	0.0121	-0.41	37
	K	0.3324	0.16	0.0144	0.40	0.0001	-0.59	37
	Ca	0.0758	0.30	0.0266	0.36	0.0003	-0.56	37
	Sr	0.0266	0.36	0.0237	0.37	0.0003	-0.56	37
	Ba	0.8923	-0.02	0.0376	-0.34	0.0014	0.50	37
